# A phase II study of oxaliplatin with 5-FU/folinic acid and concomitant radiotherapy as a preoperative treatment in patients with locally advanced rectal cancer†

**DOI:** 10.2349/biij.7.4.e25

**Published:** 2011-10-01

**Authors:** I Chitapanarux, T Chitapanarux, E Tharavichitkul, S Mayurasakorn, P Siriwittayakorn, S Yamada, V Lorvidhaya

**Affiliations:** 1 Division of Therapeutic Radiology and Oncology, Faculty of Medicine, Chiang Mai University, Thailand; 2 Division of Gastroenterology, Faculty of Medicine, Chiang Mai University, Thailand; 3 Division of Gastrointestinal Surgery and Endoscopy Unit, Faculty of Medicine, Chiang Mai University, Thailand

**Keywords:** 5-fluorouracil, folinic acid, oxaliplatin, chemoradiotherapy, phase II study, advanced rectal cancer

## Abstract

**Objective:**

To evaluate the activity and safety of adding oxaliplatin to a standard chemoradiotherapy schema, including 5-fluorouracil (5-FU)/folinic acid (FA), in locally-advanced rectal cancer (LARC).

**Methods:**

Two cycles of oxaliplatin 130 mg/m^2^ plus FA 20 mg/m^2^ bolus for 5 days and 5-FU 350 mg/m^2^ continuous infusion for 5 days were given during week 1 and 4 of pelvic radiotherapy 46 Gy. Patients with a T3/4 and/or node-positive rectal tumour were eligible. Surgery was performed 4–6 weeks after radiotherapy. The primary endpoint was to determine the rate of pathological response. Secondary endpoints were to assess the rate of clinical response and the safety profile.

**Results:**

Between March 2005 and January 2009, a total of 35 patients were enrolled. The pathological down-staging rate was 79% with a pathological complete response rate of 17%. The overall clinical response rate (assessed by computed tomography or transrectal ultrasound) was 77%. Grade 3 diarrhoea and Grade 3 neutropaenia were reported in 14% and 11% of the patients, respectively. Eleven patients did not undergo surgery: four of them refused the operation, and seven patients were inoperable due to disease progression. In 24 patients who had surgery, a sphincter-preserving procedure could be performed in 29%. At the median follow-up time of 28.1 months, 25 patients (71%) survived with no evidence of disease.

**Conclusion:**

The promising results in terms of pathological response, and the associated good safety profile of a regimen of oxaliplatin plus 5-FU/FA with concomitant radiotherapy, suggest that the regimen could be used in LARC.

## INTRODUCTION

Colorectal cancer (CRC) is the third most common cancer in males and the fifth most common cancer in females in Thailand. Its estimated incidence rate in Thailand is 8.8 for males and 7.6 for females [1], with more than 8,000 new CRC patients reported in 2008 [2]. In Thailand, because of socioeconomic problems and healthcare disparities, patients not included in the medical care system frequently have large tumour volumes and are diagnosed at an advanced stage. For example, of cases with known stage in Chiang Mai, only 10.4% were diagnosed with localised disease, whereas 29.2% had metastatic disease [1]. Progression of locally-advanced disease, with or without distant metastasis, leads to the disabling symptoms and high mortality associated with unresectable rectal cancer [3]. Hence, early and aggressive management of CRC is warranted for locoregional control and palliation of symptoms.

5-fluorouracil (5-FU) has been the mainstay of chemotherapy for CRC for almost 40 years [4], and is used both in adjuvant and palliative settings [5]. A number of biochemical modulators have been used in combination with 5-FU in an attempt to improve its efficacy while maintaining acceptable toxicity. Among them, the most successful to date is folinic acid (FA; also called leucovorin [LV]) [6, 7], and regimens based on 5-FU + FA have become standard in many institutions worldwide.

Oxaliplatin is a third-generation platinum derivative, which, when combined with 5-FU and LV, is among the most effective chemotherapies for metastatic CRC [5, 8–10]. In a pivotal phase III study, the addition of oxaliplatin to 5-FU plus FA doubled the response rate (RR) and prolonged progression-free survival among patients with metastatic CRC [5]. Oxaliplatin is registered in Europe and in the USA for the treatment of advanced or metastatic CRC, in combination with 5-FU and FA/LV. The indication encompasses both first and second -line treatment.

In the treatment of rectal cancer, concurrent chemoradiation with 5-FU improves both local control and overall survival (OS) when compared with radiotherapy (RT) alone. Thus, combined-modality treatment has been accepted as an integral part of the pre- and post-operative treatment of rectal cancer [11, 12]. In a randomised German study comparing pre- and post-operative 5-FU concurrent with radiation in rectal cancer, no significant differences between the two approaches were reported in terms of OS. However, treatment compliance, grade 3/4 toxicity, tumour down-staging, rate of sphincter preservation and rates of pelvic recurrence all favoured the pre-operative chemoradiation arm [13]. Thus, preoperative chemoradiotherapy (CRT) is clearly preferred when tumour shrinkage is required before surgery, that is, in locally-advanced T4 disease and low-lying tumours when sphincter preservation is attempted [13–15].

At present, in patients with locally-advanced rectal cancer (LARC), pre-operative CRT is the standard treatment, with 5-FU ± folinic acid being the standard chemotherapy regimen concurrent with RT. Oxaliplatin is an ideal candidate for inclusion in CRT regimens because of its radiosensitising capabilities, synergy with fluoropyrimidines [16], and its relative lack of acute dose-limiting adverse effects when added to RT and 5-FU [17]. Phase I studies showed pre-operative radiation concurrent with oxaliplatin and 5-FU/FA was well-tolerated in patients with rectal cancer [18]. Hence, based on this evidence, oxaliplatin combined with 5-FU/FA was chosen as the chemotherapy regimen concomitant with pre-operative radiation in this phase II clinical trial to test the activity and feasibility of this new pre-operative treatment in LARC.

The primary objective of this study was to assess the RR of concurrent pre-operative chemotherapy (oxaliplatin/5-FU/FA) plus radiation in patients with LARC. The secondary objective was to assess the safety profile and disease-free survival (DFS).

## METHODS

### Study Design

This was a prospective, single-centre, open-labeled, phase II study carried out at Chiang Mai University in Thailand. The study was conducted according to globally-accepted standards of Good Clinical Practice, and in agreement with the latest revision of the Declaration of Helsinki and local regulations. The planned duration of the entire study was 4 years. It was planned to enroll a total of 30 patients during the study period of 18 months. All eligible patients were registered prior to the start of treatment and treatment was started no later than 8 days after registration.

Pre-operative RT was given for a period of 5 weeks. Chemotherapy was administered synchronously on week 1 and 5 of radiation. After the end of treatment, patients were scheduled for surgery 4–6 weeks after completion of concurrent CRT. Post-operative patients received adjuvant chemotherapy on the discretion of the investigator. Patients were followed up every 3 months up to 2 years or death, whichever occurred earlier.

Treatment could be discontinued before the completion of treatment for the following reasons: progression of disease, serious adverse events (AEs) including inter-current illness and unacceptable toxicity which renders further treatment on protocol detrimental to thepatient, delay of more than 2 weeks for blood counts to recover or for non-haematological toxicities to improve to Grade 2 or less, at the discretion of the managing physician, at request from the patient, or due to death or loss to follow-up.

### Study Population

Adult patients (between 20 and 70 years) with histologically proven, locally-advanced (T3-4/N1-3/M0) adenocarcinoma of the rectum, with a measurable lesion, Eastern Cooperative Oncology Group (ECOG) performance status (PS) of ≤ 2 and life expectancy of more than 6 months were included in the study, if they had had no prior specific treatment for rectal cancer except biopsy, had adequate haematological function (haemoglobin ≥ 10 g/dL, absolute neutrophil count (ANC) ≥ 1.5 × 10^9^/L and platelets ≥ 100 × 10^9^/L), as well as adequate renal and hepatic functions (total bilirubin ≤ 1.25 × upper normal limits; SGOT, SGPT and alkaline phosphatase ≤ 3 × upper normal limits; and creatinine ≤ 1 × upper normal limits). Other inclusion criteria included the ability to comply with scheduled follow-up and with management of toxicity, signed informed consent from the patient or legal representative and negative urine pregnancy test (if indicated).

Patients were excluded from the study if they had metastatic rectal cancer, other tumour types than adenocarcinoma of the rectum, were pregnant or lactating, or were with reproductive potential but not implementing adequate contraceptive measures. Other exclusion criteria were: current uncontrolled infection; unresolved bowel obstruction or subobstruction; uncontrolled Crohn's disease or ulcerative colitis; current history of chronic diarrhoea; other serious illness or medical conditions; contraindication towards experimental drugs and radiation; past or concurrent history of neoplasm other than rectal adenocarcinoma, except curatively treated non-melanoma skin cancer or in situ carcinoma of the cervix; concurrent treatment with any other anti-cancer therapy; or administration of any other experimental drug under investigation concomitantly or within 4 weeks before eligibility.

### Evaluations Prior to Enrolment and During the Study

History and physical examination, assessment of performance status, weight, complete blood count, liver function test, blood urea nitrogen (BUN), creatinine and pregnancy test (if indicated) were carried out within 7 days prior to enrolment. Chest x-ray and CT scan of the whole abdomen for evaluation were done within 4 weeks prior to enrolment. Bone scan and CT scan of other parts (if clinically indicated) for exclusion of distant metastases were done within 4 weeks prior to enrolment.

History and physical examination including weight, Performance Status (PS) and vital signs were done on the first day of each week of radiation. Laboratory tests for full blood counts, BUN and creatinine were done on the first day of each week of radiation. Physical examinations and imaging studies were done 4 weeks after completion of radiation to assess clinical response. Toxicity of treatment was assessed during each clinic visit using the National Cancer Institute Common Toxicity Criteria (NCI-CTC) version 2. History, physical examination and late toxicity assessment using Radiation Therapy Oncology Group (RTOG) toxicity criteria were done during each follow-up visit.

### Study Treatment

**Figure 1:**
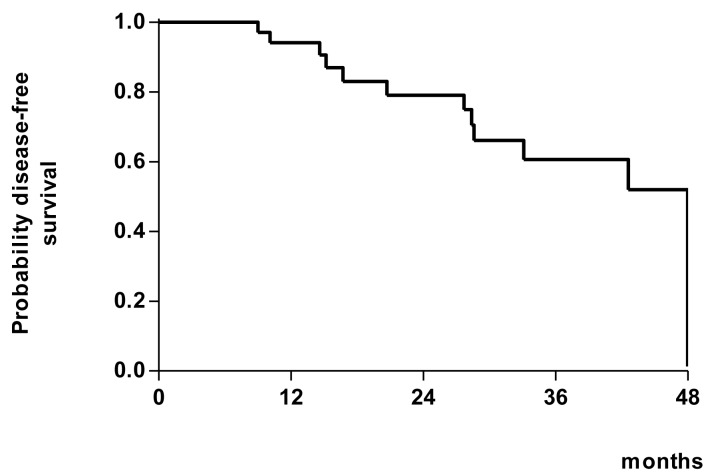
Disease-free survival.

All patients whomet the eligibility criteria underwent treatment with pre-operative chemoradiation. The treatment schema is shown in Figure 1.

#### Pre-operative evaluation for surgery

All eligible patients were evaluated for the planning of surgery before starting the treatment (between abdomino-perineal resection and sphincter-sparing surgery), and were recorded into the patient’s chart by surgeons.

#### Chemotherapy

Two cycles of chemotherapy were given synchronously on week 1 and 5 of radiation. Chemotherapy regimen started with oxaliplatin at a dose of 130 mg/m^2^ administered as a 120-minute intravenous (i.v.) infusion on day 1 followed by FA at dose of 20 mg/m^2^ i.v. bolus and 5-FU 350 mg/m^2^ continuous infusion on day 1–5 of each cycle. Anti-emetic drugs were given on the discretion of the investigator before and after chemotherapy infusion in order to prevent or palliate chemotherapy-induced nausea and vomiting.

Dose reduction was planned in case of severe haematological and/or non-haematological toxicities. Dose adjustments were made according to the system showing the greatest degree of toxicity. Toxicities were graded using the NCI-CTC version 2. If the ANC was < 1.5 × 10^9^/L, platelet count was < 100 × 10^9^/L, and diarrhoea and mucositis were NCI Grade > 1, the chemotherapy infusion was delayed until ANC recovered to ≥ 1.5 × 10^9^/L, platelet count was ≥ 100 × 10^9^/L and diarrhoea and mucositis were of NCI Grade ≤ 1. If the ANC was ≤ 0.5 × 10^9^/L (or if it was ≤ 1.0 × 10^9^/L in the presence of a fever / infection), platelet count was < 25 × 10^9^/L, and diarrhoea and mucositis were of NCI Grade 3 or 4, chemotherapy was delayed until the ANC recovered to ≥ 1.5 × 10^9^/L, platelet count was ≥ 100 × 10^9^/L, and diarrhoea and mucositis were of NCI Grade ≤ 1. Thereafter, the infusion was given with 20% reduction in doses, i.e., 5-FU 280 mg/m^2^ and oxaliplatin 100 mg/m^2^. Doses that were once reduced because of toxicity were not re-escalated.

#### Radiation

Pre-operative RT (whole pelvic RT) was given with linear accelerator machine. Treatment field consisted of anterior-posterior or four-field box technique. Conventional dose RT (200 cGy/d) was used with total tumour dose of 5,000 cGy in 5 weeks.

If the ANC was < 1.5 × 10^9^/L and platelet count was < 100 × 10^9^/L, RT was delayed for 1 week. If RT was delayed, chemotherapy was also delayed. The interval between doses was recorded. For patients who developed ≥ Grade 3 radiation proctitis, RT was delayed until the toxicity level was ≤ Grade 2. If the ANC was < 1.5 × 10^9^/L, but the platelets were > 100 × 10^9^/L, the continuing of radiation was on the discretion of the investigator.

### Prior and concomitant treatments

Every drug prescribed to the patients was recorded in the appropriate space on the case record forms. Symptomatic treatments were permitted, including any specific medication (except anti-cancer drugs) justified for any medical indication including cancer symptoms and side effects of study drugs. The patients did not receive other investigational drugs and anti-cancer treatment while on treatment.

### Study Assessments

#### Pathological response

The tumour site was entirely sectioned for pathological examination of the response of preoperative treatment. The viable tumour cells were documented as residual disease. Residual tumour was staged according to the 2002 AJCC Staging. Pathological complete response (pCR) was defined by the complete absence of tumour cells from the entire specimen.

#### Clinical response

Transrectal ultrasound and CT were performed prior to surgery. Measurable lesions were measured using the longest diameters of the lesions. Response was evaluated according to the Response Evaluation Criteria In Solid Tumors Group (RECIST) criteria at 4 weeks after complete radiation. The same method of assessment and the same technique was used to characterise each identified and reported lesion at baseline and during follow-up.

#### Disease-free survival

Follow-up continued at every 3 months after surgery for up to 2 years or death, whichever occurred earlier. Hence, the time measured from the day of eligibility to the first recurrence or death was analysed as DFS. Kaplan-Meier curve was plotted.

#### Safety profile

The following tests were performed prior to and/or on specified days during and following therapy: history of malignant and non-malignant diseases; full clinical examination, height, weight, assessment of residual toxicity due to prior therapy and disease symptoms according to NCI-CTC version 2, and PS according to ECOG criteria; and complete blood count.

Each patient was regularly assessed for potential AEs and disease-related signs and symptoms according to the NCI-CTC version 2. If not applicable, the event was graded as 1 = mild, 2 = moderate, 3 = severe, 4 = life-threatening. All events, even not related, were reported.

Withdrawal from the study and therapeutic measures were at the discretion of the investigator. A full explanation for the treatment discontinuation from the study was made on the appropriate case record form.

### Statistical Methods

#### Sample size

The main objective of this trial was to assess the clinical RR of concurrent pre-operative chemotherapy (oxaliplatin/5FU/FA) plus radiation in LARC. A RR of 60% was considered as the minimum activity level of interest, while a RR of 75% was promising. With respect to these considerations, the two-stage method of Simon’s Optimal Design was used to estimate the sample size. The parameters used were p(0) = 0.60, p(1) = 0.75, beta = 0.20, and alpha = 0.05. The sample size was calculated to be 30.

#### Statistical analyses

All untreated patients were described separately and the reasons they did not receive the treatment were given. The evaluable population consisted of eligible patients (with no major protocol violations) who had received at least one complete cycle of treatment. However, if a progression occurred before the end of the first cycle, the patient was considered as evaluable (early progression).

For the efficacy analysis, RR, and DFS were estimated. The RR, with its 95% confidence interval (using binomial estimation), was calculated on the evaluable population. DFS was analysed on the intent-to-treat (ITT) population.

Safety analysis of the population receiving treatment included analysis of AEs according to the relationship with the treatment, the severity and seriousness. Analysis by patient was provided.

## RESULTS

### Patient Characteristics

A total of 35 patients were enrolled in the study between March 2005 and January 2009. Their baseline characteristics are presented in Table 1.

**Table 1:** Baseline Characteristics.

**Characteristic**	**Value**
Age, years	
Median	54
Range	20-70
Sex, n (%)	
Male	20 (57.14)
Female	15 (42.86)
Distance between the lower margin of the tumour and the dentate line, cm	
Median	5
Range	1-13
Eastern Cooperative Oncology Group performance status, n (%)	
0	30 (85.71)
1	5 (14.29)
Clinical stage (as detected by transrectal ultrasound), n (%)	
T3N0	12 (34.29)
T3N+	17 (48.57)
T4N0	3 (8.57)
T4N+	3 (8.57)
Planned surgery, n (%)	
Abdominoperineal resection	30 (85.71)
Low anterior resection	5 (14.29)

### Response Rates

After pre-operative CRT, surgery was performed in 24 (69%) of 35 patients. The median interval from completion of radiotherapy to surgery was 6.2 weeks (range 5.5–7.5). Of these, 17 (71%) underwent abdominoperineal resection (APR), whereas 7 (19%) underwent low anterior resection (LAR). Surgery was not performed in 11 (31%) patients: in 7 cases due to disease progression and unresectable tumour and in 4 cases due to patient refusal. Sphincter preservation rate was 29%.

### Clinical Response Rates

In the intention to treat population (n = 35), 27 patients (77%) had objective response (4 complete response; CR and 23 partial response; PR), and 7 patients (20%) had progressive disease; PD. Among patients with PD, metastases were found in the liver in 2 cases, in the small bowel in 2 cases, in the bladder in 2 cases, and there was peritoneal seeding in 1 case.

### Pathological Response Rates

Of the 35 patients enrolled, 24 underwent surgery and the pathological response rate is presented in Table 2. Four in 24 patients (17%) had pathological complete response, while 19 out of 24 patients (79%) had pathological partial response. One patient had pathological stable disease.

**Table 2:** Response Rates.

**Response**	**Patients In Whom Definitive Surgery Was Performed**^1^	**Patients In Whom Definitive Surgery Was Not Performed**^2^
Complete Response	4	0
Partial Response	19	4
Stable Disease	1	0
Progressive Disease	0	7

1 response based on histopthologic assessment

2 response based on endoscopic ultrasound or CT examination

### Toxicity

The safety of the regimen was evaluated in all 35 patients. The regimen was well tolerated.

Diarrhoea was the most common non- haematologic toxicity (14%). RT was completed to the planned dose of 50 Gy. However, only one patient (3%) received delayed in the RT treatment due to grade 3 ANC. Chemotherapy was also delayed in this patient. Grade 3 leucopaenia was found in 11%. All the cases received 2 cycles of chemotherapy with RT. No grade 4 toxicity was seen during chemoradiation. Details are presented in Tables 3 and 4. There were no deaths related to toxicity during chemoradiation. No surgical morbidity was reported in 24 patients who underwent surgery.

**Table 3:** Common Haematological Toxicity (NCI-CTC v 2.0) (n=35)

**Haemato-toxicity**	**Grade 1** **n (%)**	**Grade 2** **n (%)**	**Grade 3** **n (%)**	**Grade 4** **n (%)**
Leucopaenia	4 (11)	1 (3)	4 (11)	-
Thrombocytopaenia	1 (3)	1 (3)	-	-
Anaemia	1 (3)	2 (6)	-	-

**Table 4:** Common Non-Haematological Toxicity (NCI CTC v 2.0) (n=35)

**Non-haematological Toxicity**	**Grade 1 n (%)**	**Grade 2 n (%)**	**Grade 3 n (%)**	**Grade 4**
Diarrhoea	1(3)	2(6)	5(14)	-
Nausea -vomiting	5(14)	-	-	-
Peripheral neuropathy	1(3)	-	-	-

### Follow up

The median follow-up duration was 28.1 months (range: 8–48.1 months). At the time of analysis in May 2010, there was no evidence of disease in 25 (71%) patients, 9 patients (26%) were living with disease, while 1 patient (3%) was dead.

## DISCUSSION

In this phase II study to assess the activity and feasibility of oxaliplatin + 5-FU/FA concomitant with preoperative RT in LARC, the overall clinical RR (assessed by CT or transrectal ultrasound) was 77%. The pathological down-staging rate was 79%, indicating good efficacy of the study regimen. Thus, the regimen of oxaliplatin + 5-FU/FA concomitant with pre-operative RT was found to be active in patients with LARC. It was also quite well-tolerated, and hence is a feasible chemotherapy regimen to be used in pre-operative combined-modality treatment.

The addition of oxaliplatin to standard CRT seems to be associated with a high pathological CR (pCR) [19]. Pathological CR is a reliable and reproducible surrogate for tumour response and is linked to improved outcome [20, 21]. Although achievement of a pCR is not the primary goal of neoadjuvant therapy, it has become a commonly used endpoint in many phase II trials aiming to improve the efficacy of rectal cancer treatment [16]. A pCR between 10% and 30% has been observed with combined pre-operative chemotherapy and RT protocols [16]. In several phase II trials of 5-FU, LV and oxaliplatin in pre-operative CRT, a pCR of 14% to 19% was reported [22–24]. The pCR of 17% observed in this reported study is in line with other studies using oxaliplatin + 5-FU/FA concomitant with RT.

Diarrhoea (14%) and leucopaenia (11%) were the only toxicities of Grade 3. No toxicity of Grade 4 was reported. Single-centre or limited institutional studies evaluating the combination of oxaliplatin and a fluoropyrimidine with RT for patients with rectal cancer have not reported high toxicity rates [19]. In several phase II trials of 5-FU, LV and oxaliplatin in pre-operative CRT, Grade 3/4 diarrhoea was observed in 8% to 30% patients. [22–24] Grade 3/4 leucopaenia was reported in 9% patients [23]. Hence, the toxicity profile of our study regimen was similar to that reported in other CRT regimens of 5-FU/LV/oxaliplatin. Comparing these results with the results of 5-FU concurrently to radiotherapy , there were no differences in efficacy and toxicity (grade 3 or more toxicity: 15–25%) [14, 15]. However, the incidence of acute toxicity during concurrent capecitabine and radiotherapy was lower than our results, and grade 3 toxicity was found in only 8–11% [16, 25].

After a median follow-up of 28.1 months, there was no evidence of disease in 71% of the patients. The 2-year disease-free survival was 74.9%, as shown in Figure 1. Adverse effects due to RT, such as defecation problems, occurred after a longer follow-up period [25].

The RR, or downsizing or down-staging as it can be analysed on the operative specimen, is a good endpoint to evaluate the efficacy of a pre-operative RT or CRT approach [26]. This endpoint heavily depends on the pathologic technique used to analyse the operative specimen. The rate of pCR also closely depends on the number of sections performed and the quality of search for residual cancer cells. The definition of viable cells can also be a source of discrepancy [22].

In conclusion, the good safety profile of the regimen of oxaliplatin + 5-FU/FA concomitant with pre-operative RT, associated with promising results in terms of pathological response, suggests that the regimen could be used in LARC and developed in future multi-centre phase III studies involving a larger study population.
